# Enabling Large-Scale Design, Synthesis and Validation of Small Molecule Protein-Protein Antagonists

**DOI:** 10.1371/journal.pone.0032839

**Published:** 2012-03-12

**Authors:** David Koes, Kareem Khoury, Yijun Huang, Wei Wang, Michal Bista, Grzegorz M. Popowicz, Siglinde Wolf, Tad A. Holak, Alexander Dömling, Carlos J. Camacho

**Affiliations:** 1 Department of Computational and Systems Biology, University of Pittsburgh, Pittsburgh, Pennsylvania, United States of America; 2 School of Pharmacy, University of Pittsburgh, Pittsburgh Pennsylvania, United States of America; 3 Max Planck Institute of Biochemistry, Martinsried, Germany; University of South Florida, United States of America

## Abstract

Although there is no shortage of potential drug targets, there are only a handful known low-molecular-weight inhibitors of protein-protein interactions (PPIs). One problem is that current efforts are dominated by low-yield high-throughput screening, whose rigid framework is not suitable for the diverse chemotypes present in PPIs. Here, we developed a novel pharmacophore-based interactive screening technology that builds on the role anchor residues, or deeply buried hot spots, have in PPIs, and redesigns these entry points with anchor-biased virtual multicomponent reactions, delivering tens of millions of readily synthesizable novel compounds. Application of this approach to the MDM2/p53 cancer target led to high hit rates, resulting in a large and diverse set of confirmed inhibitors, and co-crystal structures validate the designed compounds. Our unique open-access technology promises to expand chemical space and the exploration of the human interactome by leveraging in-house small-scale assays and user-friendly chemistry to rationally design ligands for PPIs with known structure.

## Introduction

Last year, the number of new drug applications (NDA) was just 18. This number poorly compares with more than 40 during the late 90's, i.e, before mapping the human genome. This reality defies all expectations that genetic research and our understanding of disease were going to lead to a new era of discoveries of novel therapeutics. Indeed, a recent analysis has shown that more than 75% of protein research still focuses on the 10% of proteins that were known before the human genome was mapped [1]. The effect of this bias has a profound effect on drug discovery, as exemplified by the popular kinase target [2]. Interestingly, the preconception that research might have somehow identified the most important proteins is also false. Instead, the origin for this bias has been traced back to the availability of small molecular weight probes for only a narrow set of targets [1]. To break this vicious circle, a new approach that stops our dependence from old compounds, and that benefits from the vast amount of information we now have on protein interactions, their structures and related diseases – *sic* system biology – is desperately needed.

The success of both high-throughput screening (HTS) and virtual screening depends on the content of the screened compound library. Since existing libraries are historically biased towards previous drug discovery efforts, the success of screening is highly correlated to traditional targets [2], [3], [4]. The latter explains in part the low hit rate of HTS when targeting new classes of proteins [3], [5], [6], [7], [8], whose chemotypes are poorly represented in current libraries [9], [10]. A promising alternative pathway is the development of suitable chemical libraries that in combination with structure-based virtual screening can significantly increase hit rates to 20% or more [4], [11], [12]. The challenge, however, is how to design large virtual selective compounds for a given target without running into the lengthy multi-step chemical synthesis that can be one of the most critical bottleneck to the chemical biology paradigm. Equally importantly is also how to bring these abstract constructs into a useful format that can leverage the ingenuity of a researcher expert on a given PPI and small-scale in house assays that today are mostly underutilized in the development of novel chemical probes of protein function.

We present a general solution to this problem by virtually designing chemically accessible compounds capable of targeting a broad set of protein-protein interactions (PPIs), a major problem in modern drug discovery [10]. Instead of focusing in virtual compounds that are often difficult to synthesize, our pipeline leverages the combinatorial chemistry of a database of known and proven (“one-pot”) chemical reactions to significantly expand the space of drug-like compounds [13]. Computational chemistry tools allow us to bias the design of the small molecules to target key anchor residues [14] for almost any protein-protein interaction with known structure. Moreover, we developed *AnchorQuery*, a user-friendly “google-like” technology capable of mining in seconds millions-size novel libraries of screening-ready products to enable a fast and inexpensive approach for pharmaceutical intervention of a myriad of known targets. We apply our technology to the MDM2/p53 cancer target, resulting in the largest and most diverse set of inhibitors to this interaction. Crystal structures of our compounds demonstrate that anchor-bound docked models significantly enhanced the quality of the predictions, strongly supporting our interactive approach to drug design.

## Results

### Expanding chemical space using multi-component reactions

**Figure 1:**
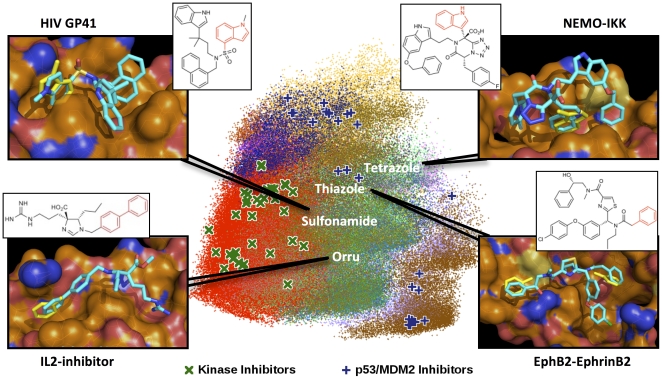
Representation of the chemical diversity of our multi-component reaction aromatic-biased libraries (different chemotypes shown in different colors) relative to the ZINC database [**20**] (red dots) and four predicted ligands. The diversity space is visualized by plotting the top two principal components of the OpenBabel FP2 (http://openbabel.org) fingerprints of 200,000 compounds randomly selected from the 17.5-and-16 million compounds of ZINC and our aromatic-biased database, respectively. The PPI-biased compounds are focused on a different region of chemical space than the historically-biased ZINC database. Indeed, a library of kinase inhibitors, some containing a tryptophan analog, falls squarely in the space covered by ZINC, while inhibitors of p53/MDM2, including inhibitors without a tryptophan analog, are located in the space covered by the new libraries. Four novel compounds from four distinct scaffolds are found to match anchors on the GP41 dimer [38], IKK [39], IL-2 [40] and EphB2 [41] receptors. Complete reaction chemistries of the *AnchorQuery* libraries can be found at http://anchorquery.ccbb.pitt.edu.

Multi-component reaction chemistry (MCR) [15] is an efficient “one-step, one pot” class of reactions that yield highly complex, drug-like and screening-ready products. Although not common in existing compound libraries, MCR compounds are well represented among known small-molecule PPI inhibitors [16], [17], [18]. More importantly, MCR derived peptido-mimetic chemotypes allow us to design compound libraries that include chemical mimics of key amino acids important for molecular recognition [19]. For instance, using 23 MCR chemistries and a curated set of commercially available or easily accessible starting materials, we have designed anchor-biased libraries of >5 million compounds targeting phenylalanine, tyrosine, tryptophan and valine/leucine, adding to more than 21 million compounds, where every compound contains a chemical analog of the targeted amino acid (see Methods). A diversity analysis that compares the 16 million aromatic-biased compounds and the 17.5 million compounds of the ZINC database [20] shown in **Figure 1** confirms that these MCR compounds encompass an untapped region of chemical space that is a departure from historical targets, such as kinase inhibitors, while amenable to PPI targets, such as the p53/MDM2 interaction. These libraries, which already match the number of commercially available drug-like compounds (see http://anchorquery.ccbb.pitt.edu/reactions/) are available for screening and download.

### Design of Anchor-biased libraries

The inclusion of amino acid analogs allows us to design libraries for specific “druggable” sites. To leverage this feature, we benefit from the growing structural information on protein-protein interactions exemplified by the Protein Data Bank (PDB) and the validated binding sites revealed by co-crystals of PPIs. The physicochemical characteristics of these interfaces have so far proven to be very challenging for drug discovery: contact surfaces involved in protein–protein interactions are typically large (1,500–3,000 Å^2^) and flat [21], and only a few success stories have been reported (e.g., Bcl2 [22], (X)IAP [23], and p53/MDM2 [24]). However, a common element of several of these compounds is specific moieties that mimic amino acid side chains of the donor protein that are found deeply buried in the acceptor protein. These anchor motifs often play a critical role in molecular recognition [14], [25], [26] by targeting relatively stable surface pockets on the receptor. Lacking biochemical mutational data, anchor side chains correlate with those that bury the largest amount of solvent accessible surface area upon binding [14]. Online tools are available to search the PDB for anchors [27], revealing thousands of potential druggable protein-protein interactions that are “biased” to the known chemistry of these key residues. A PDB-wide statistics (see **Figure S1**) shows that aromatics and leucine are the most enriched class of anchors among all interface residues in PPIs. Thus, our motivation for designing PPI-biased libraries of compounds containing specific analogs of Phe, Tyr, Trp, or Leu/Val residues, as a first step towards being able to selectively target PPIs in the PDB.

### *AnchorQuery*: the first web-based technology for rational drug discovery

In parallel with the development of million-size anchor-biased libraries, we have developed an anchor-oriented virtual screening technology to facilitate the *rational design* of small molecule protein-protein antagonists. The goal of virtual screening is to generate a substantially reduced and enriched subset of compounds from a virtual chemistry space. Similarity search methods, despite their simplicity, have been shown to be remarkably effective [28], but are less applicable when screening for PPI inhibitors since there are few described active ligands. Docking, which positions and scores ligands within the interaction interface, can also be effective [29] and provides useful structural insight, but is computationally demanding. Another approach is a pharmacophore search, an established mechanism for virtual screening that matches essential features of ligands with derived or predicted features of an interaction (see review by Leach et al. [30]). Our method is a novel implementation of all the above, where we integrate the similarity of the protein ligand into our biased libraries, the efficient docking of the anchor-analogs into the anchor of the PPI and the direct matching of pharmacophores (i.e., hydrophobic, hydrogen bonds, and aromatic rings) into the docked models.

The technology, referred to as *AnchorQuery*, performs an exact pharmacophore search of anchor-oriented virtual libraries of explicit conformations. The computational performance of most pharmacophore search implementations is directly proportional to the size of the database limiting the effective size of virtual libraries. *AnchorQuery* pharmacophore search uses a specialized spatial index [31] so that searches scale with the breadth and complexity of the query, not the size of the database. Unlike previous methods, *AnchorQuery* does not require a highly reduced chemical space [32] nor is it limited to specific chemical scaffolds [33].

**Figure 2:**
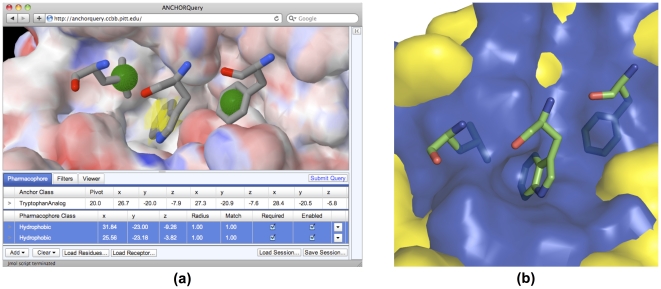
The p53/MDM2 protein-protein interaction and open-access *AnchorQuery* web interface. (a) A screenshot of the online *AnchorQuery* software show a query pharmacophore for targeting the p53/MDM2 interface. Two hydrophobic features with a 1 Å radius (green) are extracted from the F19 and L25 amino acids of p53 while a Tryptophan analog pharmacophore feature (yellow), unique to *AnchorQuery*, matches the indole fragment of tryptophan. (b) In the p53/MDM2 complex (PDB 1YCR) three residues (F19, W23, L25) of p53 (green sticks) are buried in MDM2 (yellow surface). Only the W23 pocket is apparent in the unbound structure (PDB 1Z1M), suggesting that W23 plays a major role in molecular recognition.

*AnchorQuery* is provided as an *open-access*, full-featured web server at http://anchorquery.ccbb.pitt.edu. The goal of this technology is to maximize the involvement of experts in collaborative protein-based chemical biology design projects. The tool is readily available to researchers around the world, enabling large-scale design and synthesis of novel compounds suitable to interfere specific PPIs. Pharmacophore features are automatically identified and can be edited within the graphical interface, shown in **Figure 2(a)**. Additional information and a user guide are provided at the website. Anew searches of billion conformations are computed in a matter of seconds (see, e.g., online Interactive Examples: Il-2, Caspase9, GP41, Ikk, and EphB2). Screens can further prune the number of hits by setting a maximum number for hits per compound, pharmacophore matches, or molecular weight. The list of hits satisfying the query is ranked according to several possible criteria: pharmacophore matches, pharmacophore RMSD, number of rotatable bonds, MCR scaffold or molecular weight (MW). The user interface includes Jmol (http://jmol.org) based visualization of pharmacophore aligned or energy minimized hits. Given that a key goal is to fast track the development of novel chemical probes, all 21+ million compounds are linked to their detailed synthesis protocols available by simply clicking on the reaction of each hit.

### Application to p53/MDM2

The tumor suppressor p53 and its negative regulator MDM2 is one of the most intensely studied PPIs, with a small number of potent small molecular weight antagonists [17], [24], [34]. The co-crystal structure [35] reveals that Trp23 of p53 is the most buried side chain on MDM2 and a natural site to validate our technology (**Figure 2(b)**). Interestingly, the NMR structure of the unbound receptor highlights both the plasticity of the binding interface and the fact that the cavity of Trp23 is more stable than that of any other buried group. The latter strongly supports a rational anchor-based approach for developing PPI antagonists. An example of a p53/MDM2 pharmacophore defined within *AnchorQuery* is shown in **Figure 2(a)**.

*AnchorQuery* has been validated as part of a drug discovery effort targeting the anti-cancer p53/MDM2 PPI. As a proof of principle, we screened compounds from a prototype library of 20 MCR reactions (**Table S1**; see Methods). The iterative screening of several variations of the core pharmacophores shown in **Figure 2(a)** successfully identified a broad set of MCR-antagonists representing different scaffolds and starting materials (see **Fig. 1**). The screens employed a variety of designs that aimed at filling the hydrophobic pockets, keeping a low molecular mass, and, exploiting the diversity of scaffolds (see Discussion). We synthesized and validated using fluorescence polarization and NMR, 80 inhibitors (<60 µM binding activity) of p53/MDM2 that contain an indole tryptophan anchor analog (some compounds were found in smaller targeted screenings [12], [36]). Additionally, we derived 13 MDM2 antagonists from a novel acyclic scaffold that was highly enriched using the pharmacophore shown in **Figure 2(a)** (see below). *The identification of these active compounds is a direct validation of the utility of AnchorQuery*.

### Refinement and validation of an *AnchorQuery*-based virtual screen

**Figure 3:**
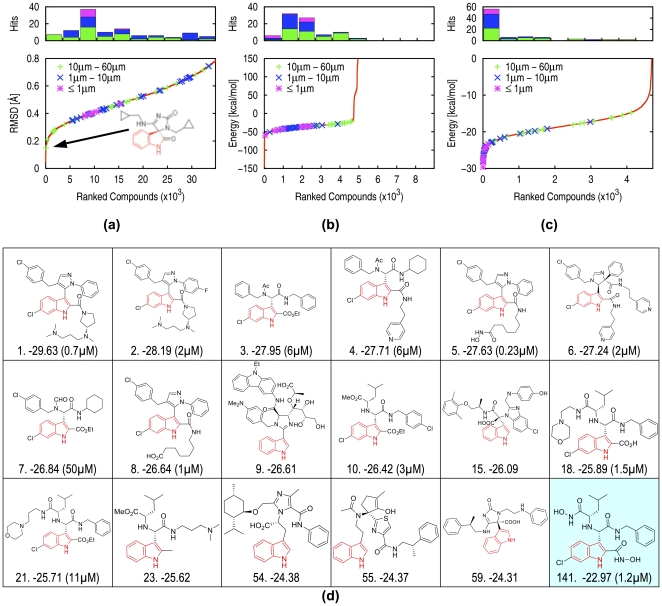
Refinement and validation of an AnchorQuery-based virtual screen. Each active compound was annotated with the binding affinity of the racemic mixture. The library (see Methods) was searched using the pharmacophore of **Figure 2**. (a) The position of active compounds of three affinity classes (points and histogram) within the RMSD ranked *AnchorQuery* pharmacophore search results (red line). Pharmacophore RMSD successfully extracts all the high affinity (sub-µM) known actives from the library. (b) A similar plot after fast minimization and pharmacophore filtering. This step effectively identifies compounds with unresolvable steric and electrostatic clashes and provides a more efficient starting configuration for the next minimization step. (c) After minimization using a Poisson-Boltzman solvent model, high-affinity compounds are noticeably differentiated from low-affinity compounds. (d) A selection of the top ranked compounds from the screen belonging to seven different scaffolds. Shown are the top ten ranked compounds, which include nine validated inhibitors, and five virtual compounds (15, 23, 54, 55, 59) selected to highlight the diversity of the results. For the K_i_ data, compounds 3, 4, 7 are measured as racemic mixtures, and compounds 10, 18, 21, 141 are measured as diastereomeric mixtures. Compound 23 was developed into a series of related compounds, including 18, 21, and 141, which is shown crystallized in **Figure 4**.

To further demonstrate the potential of *AnchorQuery* to expedite the discovery of novel compounds, we screened a Trp-biased prototype library of 600,000 compounds (see Methods). Since the library is virtual, compounds synthesized by the efficient MCR chemistry often include slight variations relative to the predicted hits due to availability (expense) and experience of the chemists with the starting materials and reactions. Hence, for the analysis presented here, we have seeded all our 93 active compounds into our Trp-biased library. We screen this library using the pharmacophores shown in **Figure 2(a)**. In a few seconds, the virtual search of all conformers (more than half a billion) led to an enriched subset of docked compounds that recovered most of our hits. In **Figure 3(a)**, we keep the three lowest pharmacophore RMSD conformational poses for each compound resulting in 77343 conformations (0.08% of the library conformations) of 34876 compounds (5.9% of the library compounds). The results include 78.5% of the known inhibitors resulting in an enrichment factor of more than 10-fold. The inset in **Figure 3(a)** shows the inhibitor with the lowest pharmacophore RMSD. This compound has a 20 µM binding affinity and belongs to a family of compounds that include sub-micromolar inhibitors [12]. Note that the visualization tool of *AnchorQuery* provides a straightforward visual validation of the pharmacophore design, and allows the user to rapidly identify scaffolds that are a good starting point for rational modifications and/or to incorporate knowledge based changes.

**Figure 4:**
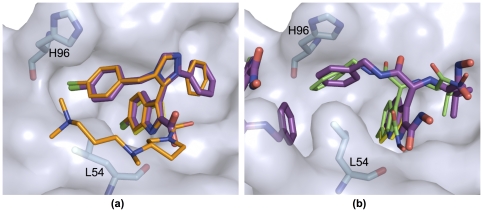
Two crystal structures of p53/MDM2 inhibitors validate the anchor-centric approach and docked models. In both structures the indole anchor analog of tryptophan overlaps almost perfectly with W23 in p53 (shown in yellow sticks), when the receptors are aligned the MDM2 structure in the co-crystal (PDB 1YCR). (a) The ligand (purple sticks) of PDB 3LBK has a very similar binding mode to the number one hit in our virtual screen (orange sticks). (b) The crystal pose of the *AnchorQuery* derived compound (purple) with the predicted pose (green) also aligned very well. The presence of a second ligand near the binding interface distorts the receptor shape relative to the receptor used for docking.

Since the main goal of *AnchorQuery* is the identification of PPI antagonists starting points (i.e., “hits”) by a rationally driven iterative process, the actual structural refinement and scoring of the virtual hits is left to the preference of the user [37]. Hence, the utility permits the free download of the designed compounds and their associated synthesis pathway for in-house optimization and synthesis. The above notwithstanding, to demonstrate the relationship between pharmacophore matching of docked ligands and a physically-based scoring function, **Figure 3** shows a multi-step energy-based refinement and ranking of the results. **Figure 3(b)** displays the re-ranking after a fast fixed-receptor energy minimization (see Methods; without solvation) of the *AnchorQuery* pharmacophore ranked compounds (**Fig. 3(a)**). This secondary screening quickly identifies complexes with clashes (i.e., interaction energy >0) resulting in a substantial enrichment of known actives in the top 5,000 compounds (an enrichment factor of more than 50-fold relative to the full library). Moreover, we verified that, after minimization, 89% of the computer generated docked structures of validated inhibitors remain close to their original pharmacophore aligned poses, suggesting that our fast and exact docked alignments yield satisfactory low-resolution models (see also **Figure 4** and **Figure S2**). The best evidence that *AnchorQuery* selects a meaningful set of compounds is shown in **Figure 3(c)**, where a detailed scoring function (see Methods) that includes solvation effects places almost all our high-affinity inhibitors in the top 150. A selection of the top scoring compounds is shown in **Figure 3(d)**. Remarkably, 9 of the top 10 chemically-distinct compounds are validated inhibitors (see **Table S2** for dose-response curves). Compound 23 in this ranking, a novel acyclic scaffold, led to the synthesis of 20 slight derivatives (the exact cpd. 23 was not synthesized). A total of 13 compounds were found to be active, six of which, including compounds 18 and 21, are ranked in the top 50 of the virtual screen. One of the most important benefits of screening anchor-enriched million-size libraries is the rich structural diversity of our hits: inhibitors from five different reaction classes are among the top 10 ranked compounds.

### New class of MDM2 inhibitors

Crystallization of MDM2 with *AnchorQuery* derived hits confirms the efficiency and usefulness of our anchor-centered approach. The two crystal structures in **Figure 4** show that both the anchor analog and the docked models superimpose well with the anchor side chain of MDM2/p53 and crystal structures, respectively, validating our premise that anchor binding sites are natural targets for pharmaceutical intervention. **Figure 4(a)** is similar to compound 1 of **Figure 3** and it has the same scaffold as the hit shown in the inset of **Figure 3(a)**. However, one of the most important and unique features of our approach is that it can identify compounds based on multiple scaffolds (chemotypes). **Figure 4(b)** shows the crystal structure of a new class of MDM2 inhibitors derived from hit 23 of *AnchorQuery*, elucidating the first MDM2 antagonist based on an acyclic scaffold. It reveals that the chloroindole ring of the inhibitor fills the tryptophan pocket of MDM2 and is stabilized by hydrophobic interactions and the hydrogen bonding with the L54-MDM2 carbonyl oxygen. The isobutyl part of the inhibitor occupies the phenylalanine pocket and keeps numerous hydrophobic interactions to MDM2. The expanded scaffold of the new class of inhibitors allows conformational plasticity and promotes an unusual stacking interaction of the compound's phenyl ring with the H96-MDM2 side-chain. This π-π interaction provides yet a new starting point for further diversifying our search for novel classes of inhibitors.

## Discussion

Due to the high costs of traditional high throughput screening (HTS) approaches [3] and often complex hit optimization chemistry, most academic researchers lack the infrastructure to embark in drug discovery efforts. Furthermore, the rigid format of HTS is not amenable to incorporate and take advantage of the biological and/or chemical insight that might exist for a given target. To leverage this know-how as well as in-house small-scale assays often available to individual researchers, we have developed *AnchorQuery*, the first web-based screening technology to rationally scrutinize different sets of hot spots to select suitable chemical probes of protein interactions for optimization and synthesis.

Our platform builds on the role anchor side chains, i.e., those that bury a large amount of surface area in the acceptor protein, have in PPIs. At the onset of the recognition process anchors target well-defined “druggable” pockets. We use chemical mimicry of these side chains to design small molecule inhibitors using fast and efficient MCR chemistry. Contrary to traditional stepwise multistep sequential synthesis, MCR assembles advanced starting materials into a new product in a “one-pot” procedure thus saving tremendous time and effort when testing the computational hypotheses. This approach has led to the development of a broad set of novel active inhibitors of MDM2 and p53 interactions, with crystal structures (**Fig. 4**) confirming the benefits of targeting the known chemistry of anchor side chains.

The success identifying compounds based on multiple scaffolds for p53/MDM2 led us to significantly expand our prototype library to maximize the potential benefits of this new PPI-biased chemical space and virtual screening technology. Currently, a curated set of 21 million virtual compounds amenable to targeting any PPI with a tryptophan, tyrosine, phenylalanine or leucine/valine anchor is online, and libraries mimicking other anchors are under development. *AnchorQuery* provides a direct link to this chemical space for the screening of any PPI that involves anchors present in our library. Applying the new libraries to p53/MDM2 (see Interactive example in *AnchorQuery*) results in an enrichment of the van Leusen scaffold of trisubstituted imidazoles. A straightforward change of the hydrophobic pharmacophores in **Fig. 2(a)**, Phe19 and Leu25, for aromatic ones leads to top hits ranked by molecular weight that are almost identical (**Fig. 4(a)**) to compounds recently shown by us [36] and researchers of NOVARTIS (Boettcher A, et al. 3-Imidazoyl-indoles for the treatment of proliferative diseases, WO 2008119741) to be highly active. More generally, **Figure 1** shows four virtual examples of interesting targets: GP41 [38] and IKK [39] with a tryptophan anchor, and IL-2 [40] and EphB2 [41] with anchors that can be targeted with our phenylalanine-biased library. The minimized compounds match the anchor (in yellow), predicting compounds that recover the chemistry seen in the co-crystals. Moreover, the server also specifies the synthesis pathway for all the compounds, a unique feature relative to other fragment-based or virtual screening technologies that are limited by chemical synthesis.

Despite the thousands of validated protein-protein interactions, the pace of discovery of chemical probes that can dissect the role of individual protein interactions in signaling pathways remains slow. Besides technical barriers, a major shortcoming in these efforts is the lack of synergy between mature disciplines like chemistry, which seeks to develop molecules with “drug-like” properties, and biology, which inquires about functional aspects of protein-protein interactions. *AnchorQuery* is a real-time, user-friendly, open-access technology that delivers more than 21 million chemically synthesizable compounds to facilitate truly integrative and interdisciplinary research. With little or no effort human insight can be incorporated into virtual screening of a novel chemical space to test assays and new discovery strategies of small molecular weight probes of protein function.

## Methods

### Library Design

We created our prototype tryptophan-biased virtual library by randomly drawing indole-containing compounds from a diverse set of 20 multi-component reactions (see **Table S1**). A total of 54,000 reactions were performed using randomly chosen reactants and ChemAxons REACTOR software (http://www.chemaxon.com). OpenEye OMEGA (http://eyesopen.com/) was used with the default settings to enumerate 591,227 stereoisomers and generate 97.9 million conformations.

Our ultimate goal is to develop libraries for all meaningful anchor amino acids. Based on our experience with the prototype library, we have currently created larger anchor-oriented libraries for phenylalanine, tyrosine, and tryptophan anchors. These libraries are created from an expanded set of 23 MCR chemistries and starting materials that are curated for affordability, diversity, and synthesizability. Complete details of the reaction chemistry are provided at http://anchorquery.ccbb.pitt.edu. We do not exhaustively enumerate all stereoisomers of compounds, instead only sampling stereoisomers around stereocenters that significantly change the geometry of the resulting conformations. 100 conformations are generated with OMEGA with a RMS cutoff of 0.7, resulting in roughly 6×10^8^ conformations per AA-based MCR library.

An analysis of typical drug-like properties of the resulting MCR libraries shows that 25% of the compounds follow all 4 of Lipinski's rules; 66% of the compounds follow 3 out of 4 Lipinski's rules; 38% of the compounds follow rule “Molecular Weight bellow 500 g/mol”; 59% of the compounds follow rule “Absolute Value of Log P≤5”; 99% of the compounds follow rule “Hydrogen Bond Acceptor ≤10”; 95% of the compounds follow rule “Hydrogen Bond Donor ≤5”; and, 58% of the compounds have “rotatable bonds ≤10”. However, we note that the concept of “drug-like” compounds in the framework of PPIs is not yet settled. Furthermore, *AnchorQuery* does not deliver optimized compounds but rather starting points for optimization, and often it is in this second stage that drug-like properties are developed.

### Pharmacophore search

The pharmacophore features are identified using standard SMARTS expressions (REF). The coordinates of a feature are determined by averaging the coordinates of all atoms matched by the SMARTS expression. The default set of pharmacophores includes expressions to match hydrogen bond donors and acceptors, positive and negative ions, aromatic rings, and hydrophobic regions (see also [31]). Pharmacophore features of a conformation are represented in the coordinate system defined by the anchor analog in the compound. These anchor-oriented features are decomposed into coordinate-frame triangles that are stored as exact coordinates in a *spatial index*, a query pharmacophore is similarly decomposed into triangles and the results of range queries on the spatial index are reconstructed into an alignment of virtual compounds to the query pharmacophore. The spatial index used by *AnchorQuery* is a custom variant of the Pharmer KDB-tree [31], which supports the efficient storage and retrieval of data indexed by spatial coordinates. This choice of data structure is particularly well suited for performing efficient range searches over point data that is stored on disk. Since all *AnchorQuery* pharmacophore queries must contain an anchor pharmacophore feature, the query features can also be represented in an anchor-oriented coordinate system and identifying all compounds that match a query feature is a simple range query in the spatial index.

### Secondary Screening

All energy minimization calculations are performed using the Merck Molecular Force Field [42] with OpenEye szybki software version 1.3.4. The results of *AnchorQuery* pharmacophore search are first quickly minimized within a fixed receptor (PDB 1YCR) with no solvent model and Coulomb electrostatics to eliminate ligand poses that are sterically or electrostatically infeasible. Minimized conformations are then filtered against the original pharmacophore. Only conformations that have a pRMSD less than one and an energy score less than zero are retained. The best scoring conformation is than further optimized within a fixed receptor using Poisson-Boltzman electrostatics. Again, minimized results that no longer match the original pharmacophore query (>1.0 Å pRMSD) are filtered out. At the transition between each stage the number of sampled conformations is reduced to match the available computing resources (the top 3 pharmacophore RMSD conformations are selected from the *AnchorQuery* results and the top conformation of each stereoisomer is selected from the first energy minimization). The screen shown in **Figure 3** took less than 12 hours on a single 3.33 Ghz Core i7 975 workstation with 12 GB of RAM.

### Chemical Synthesis

All chemicals were purchased from Aldrich, Fisher Scientific, Acros Organics or Alfa Aesar and used as received. ^1^H- and ^13^C-NMR spectra were recorded on a Bruker Avance II Ultrashield Plus 600 at 600 and 150 MHz, respectively. Chemical shift values are in ppm relative to residual solvent signal. Abbreviations used are s = singlet, brs = broad singlet, d = doublet, brd = broad doublet, m = multiplet; data in parenthesis are given in the following order: multiplicity, number of protons and coupling constants in Hz. Flash chromatography was performed with the indicated solvent mixture on silica gel, MP Silitech 32–63 D, 60 Å, Bodman. Thin layer chromatography was performed using Whatmann flexible-backed TLC plates on aluminum with fluorescence indicator. Compounds on TLC were visualized by UV-detection. HPLC-MS measurements were done on a Shimadzu prominence HPLC equipped with a dual wavelength UV detector and an API 2000 LC-MS/MS system, Applied Biosystems MDS SCIEX, (MS) using a Dionex Acclaim 120 column (C18, 3 µm, 120 Å, 2.1×150 mm) using a mobile phase of water and acetonitrile, both containing 0.1% acetic acid and the following gradient: 5–90% acetonitrile in 7 min, injection volume: 5 µL, detection wavelength 254 nm. HRMS measurements were performed at the Department of Chemistry, University of Pittsburgh with a Waters/Micromass Q-Tof spectrometer. Details of each chemical reaction can be found in the **Methods S1**.

### Fluorescence polarization binding assays (FP)

All FP experiments were performed as described by Czarna et al. [43] and are also described in the Methods S2.

### Protein expression, purification, crystallization, data collection and structure solution

See Methods S2.

## Supporting Information

Figure S1 **The distribution of the most deeply buried anchor (blue) with at least one anchor residue (ΔSASA>80Å and >70% of SASA is buried), compared with the relative frequency of each residue in proteins.**
(TIFF)

Figure S2 **Virtual docking poses of the compounds of** **Figure 3(d)** **. Compounds 1 and 141 are shown in** **Figure 4** **.**
(TIFF)

Table S1 Multicomponent reactions used in the generation of a tryptophan-biased library. These reactions, together with a set of roughly 1000 commercially available starting materials, define a theoretical chemical space of more than three trillion distinct chemical compounds. Requiring at least one indole starting material in each reaction yields as many as 190 billion compounds containing a tryptophan mimic.
(DOC)

Table S2 Inhibition curves of inhibitors from **Figure 3(d)** shown with rank and affinity.
(DOC)

Methods S1 **Chemical Synthesis.**
(DOC)

Methods S2 **Experimental Procedures and Supplementary References.**
(DOC)
