# Supplementary figures and images for: Enabling Large-Scale Design, Synthesis and Validation of Small Molecule Protein-Protein Antagonists
Source: PLoS One. 2012 Mar 12;7(3):e32839. doi: 10.1371/journal.pone.0032839 (PMC3299697; doi:10.1371/journal.pone.0032839)

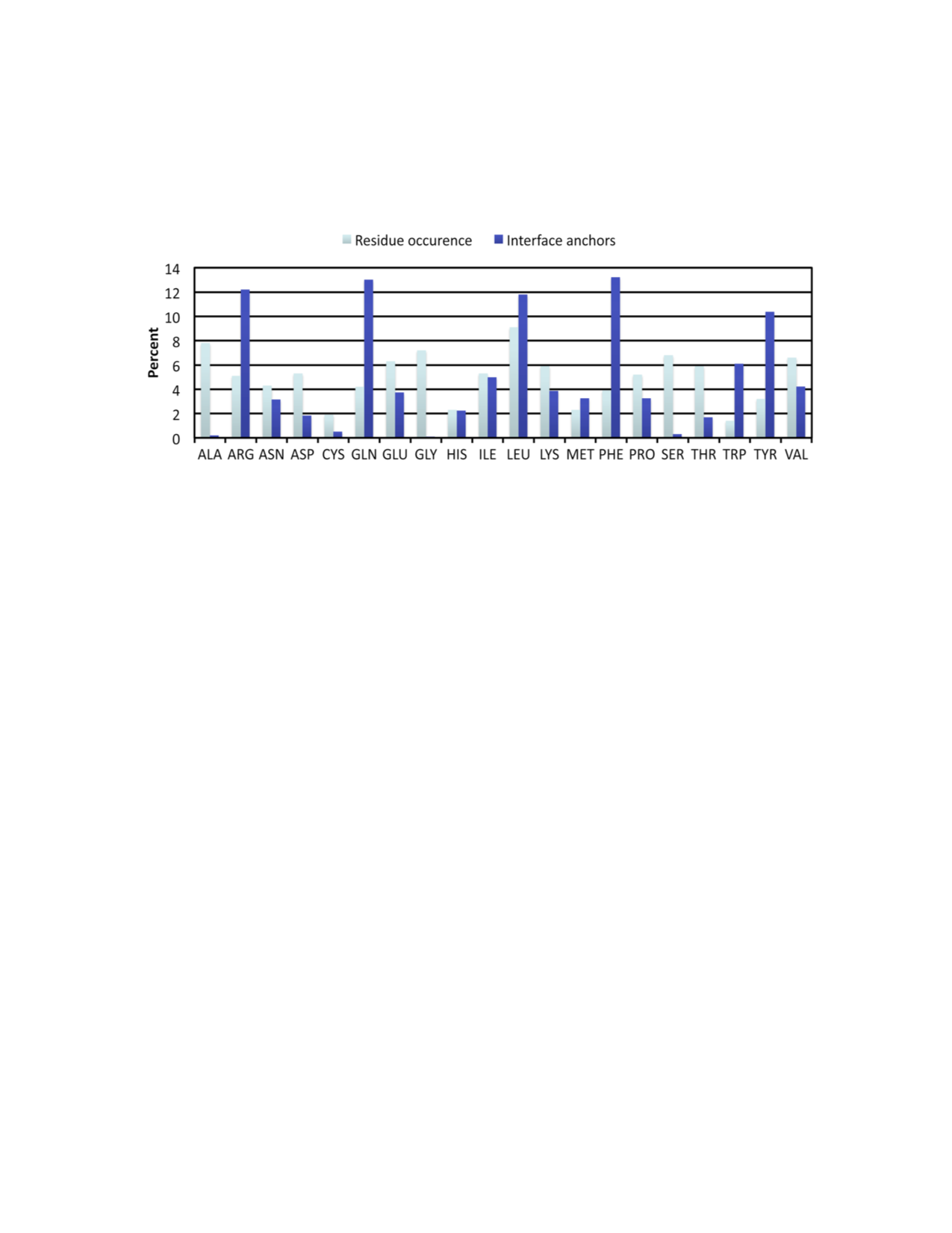

Supplement: Figure S1 — The distribution of the most deeply buried anchor (blue) with at least one anchor residue (ΔSASA>80Å and >70% of SASA is buried), compared with the relative frequency of each residue in proteins. (TIFF) [file pone.0032839.s001.tiff]

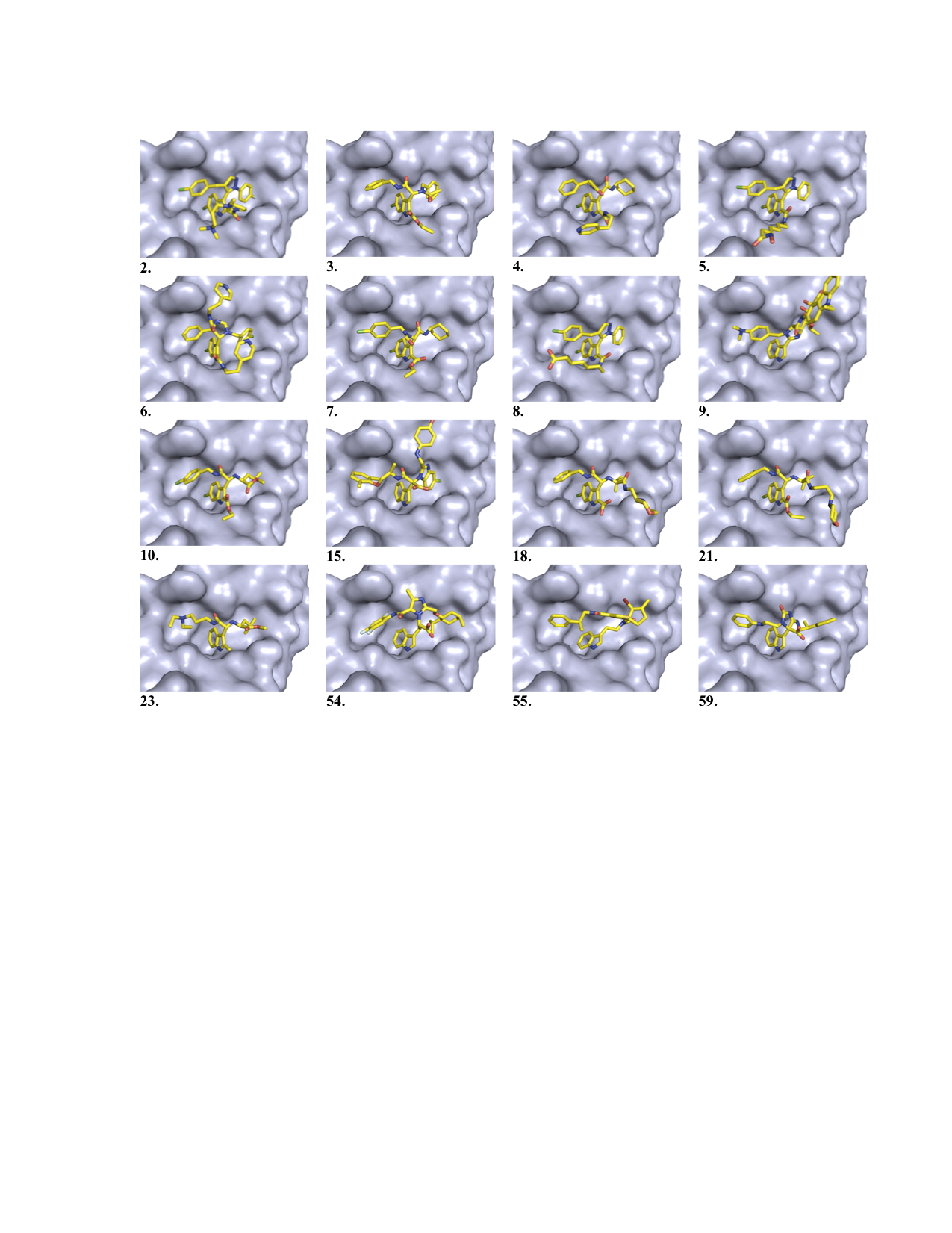

Supplement: Figure S2 — Virtual docking poses of the compounds of Figure 3(d) . Compounds 1 and 141 are shown in Figure 4 . (TIFF) [file pone.0032839.s002.tiff]

| 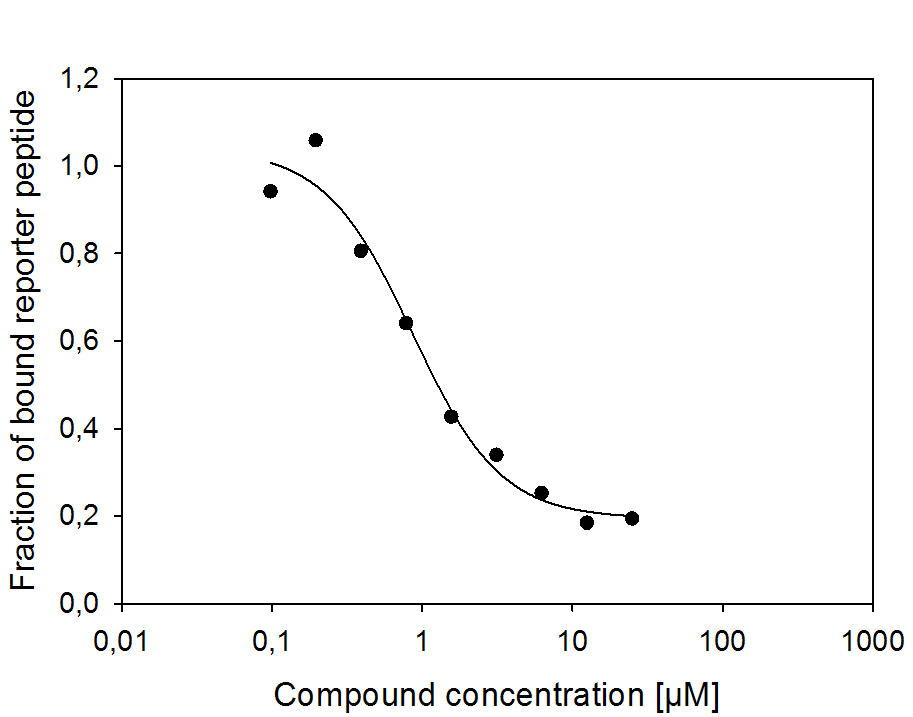  **1.** 0.7 µM | 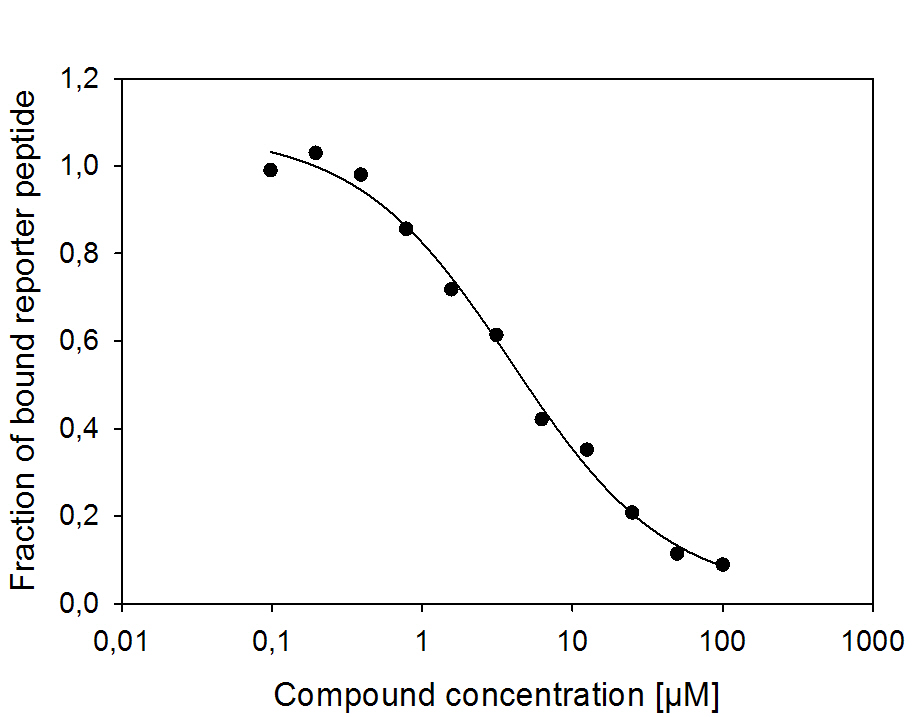  **2.** 2µM | 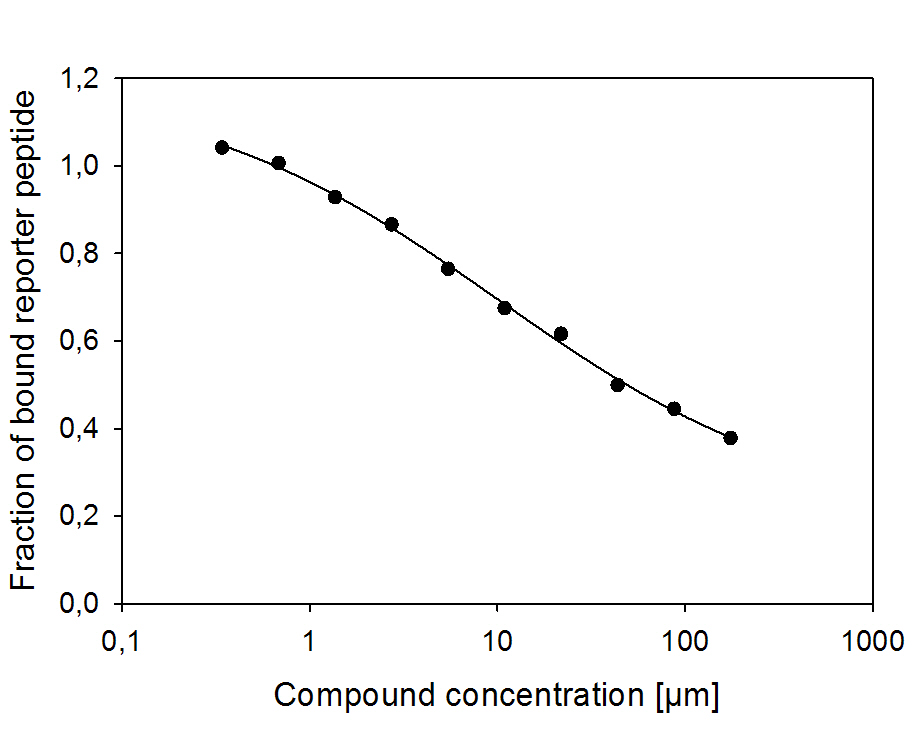  **3.** 6µM |
| --- | --- | --- |
| 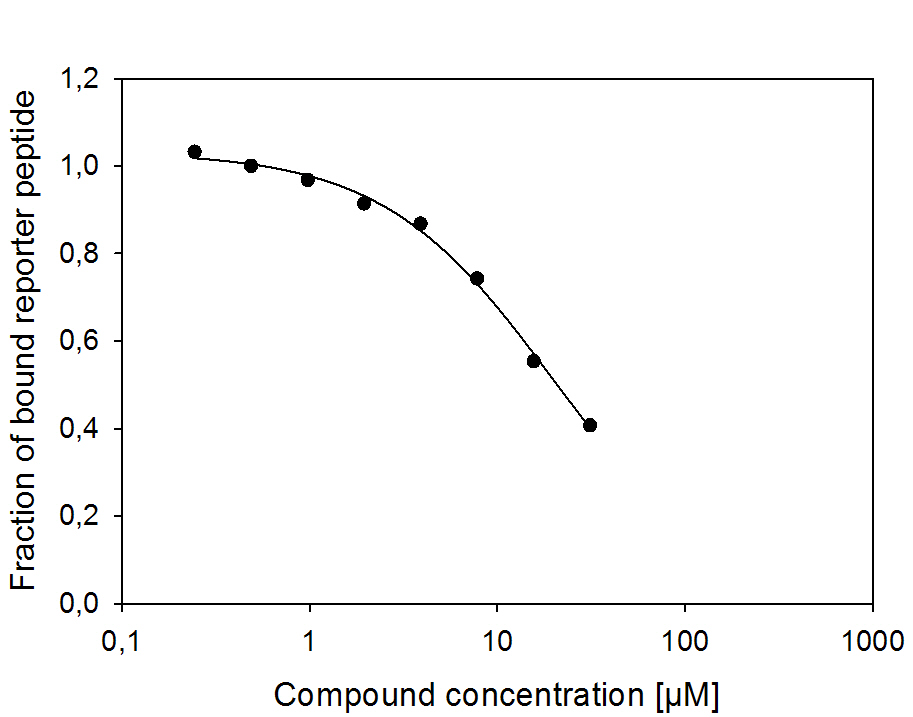  **4.** 6µM | 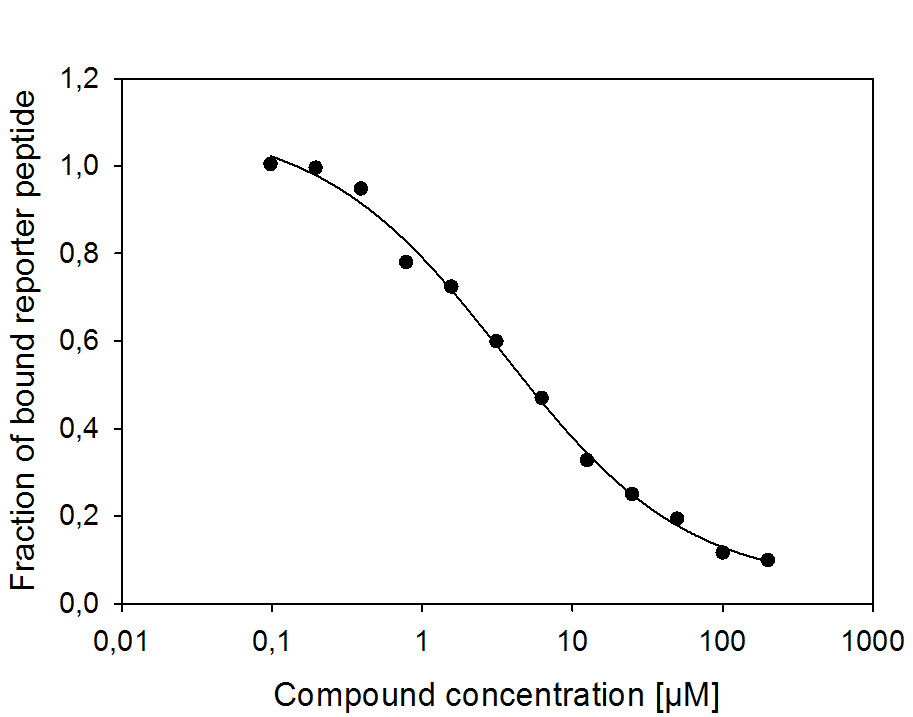  **5.** 0.23µM | 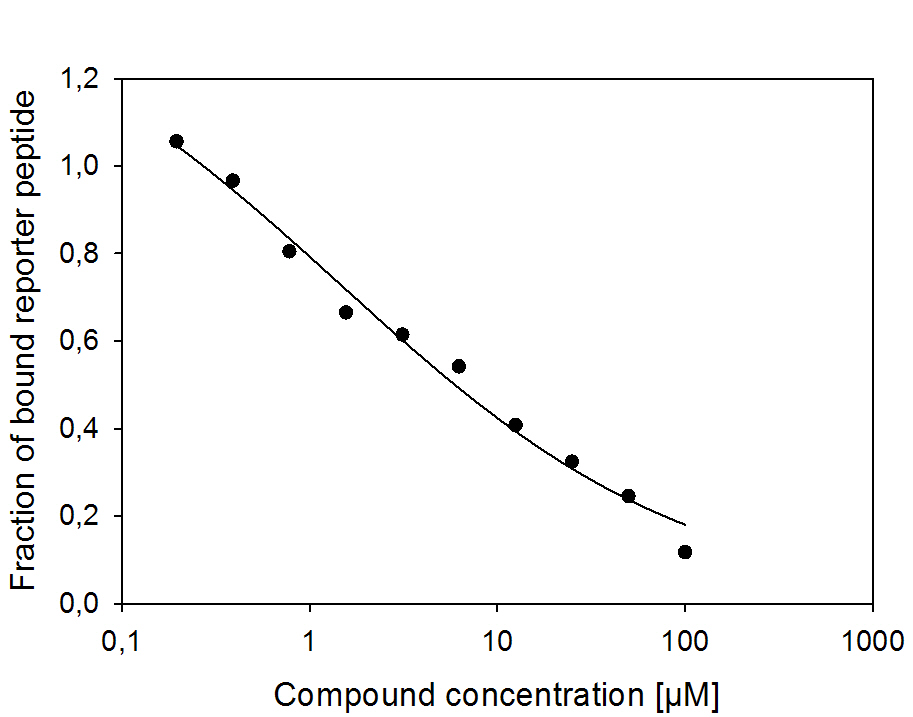  **6.** 2µM |
| 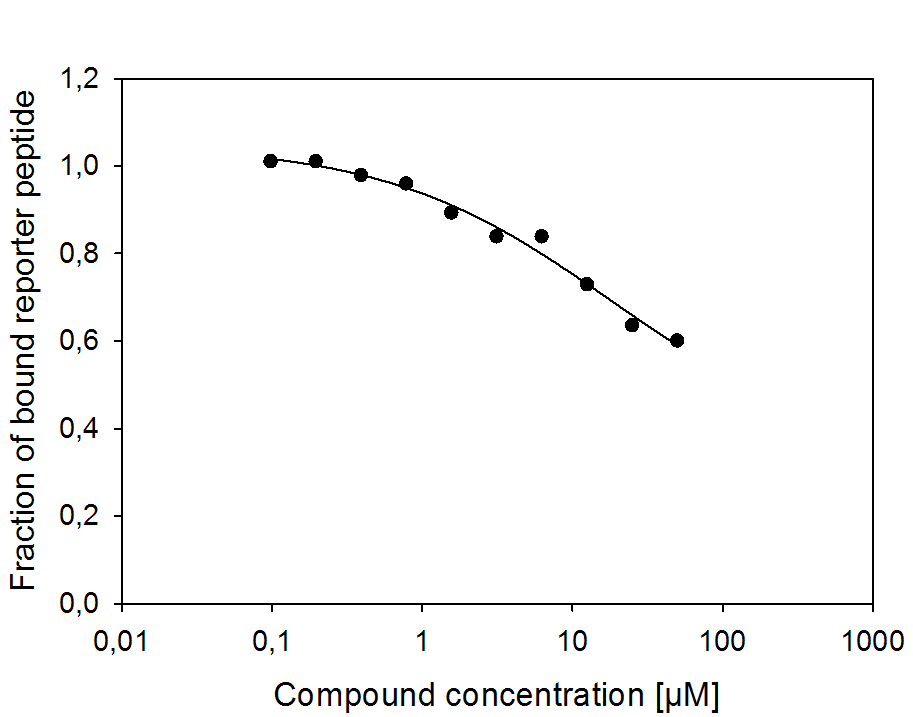  **7.** 50µM | 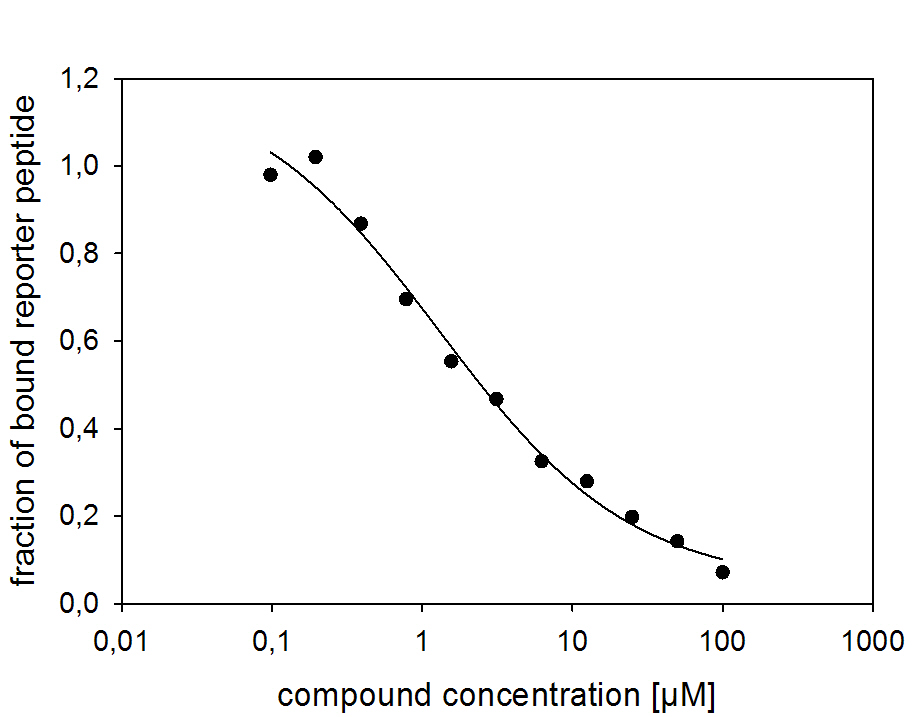  **8.** 1µM | 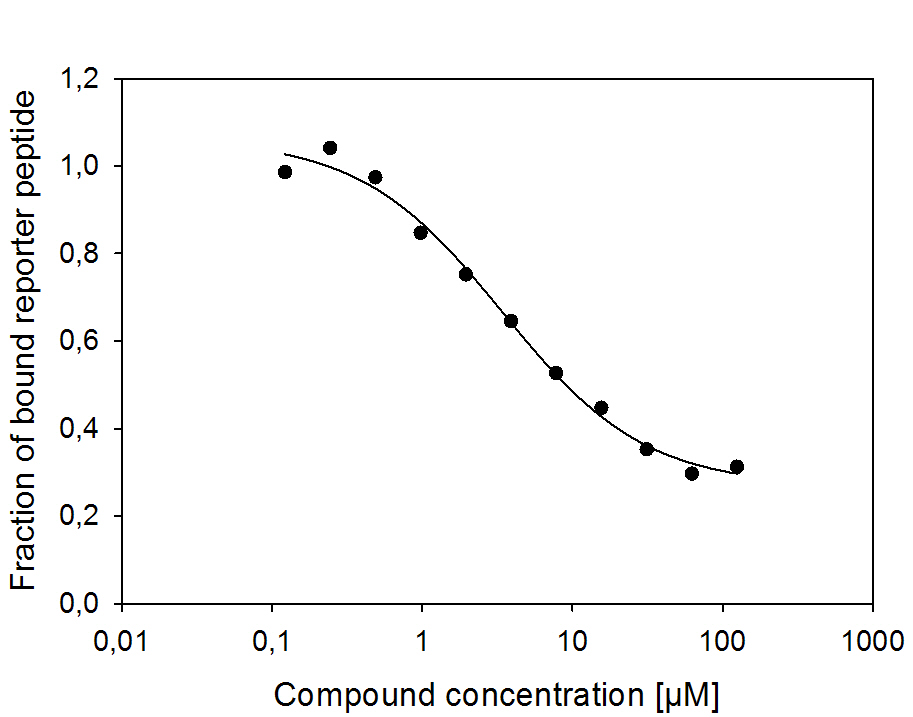  **10.** 3µM |
| 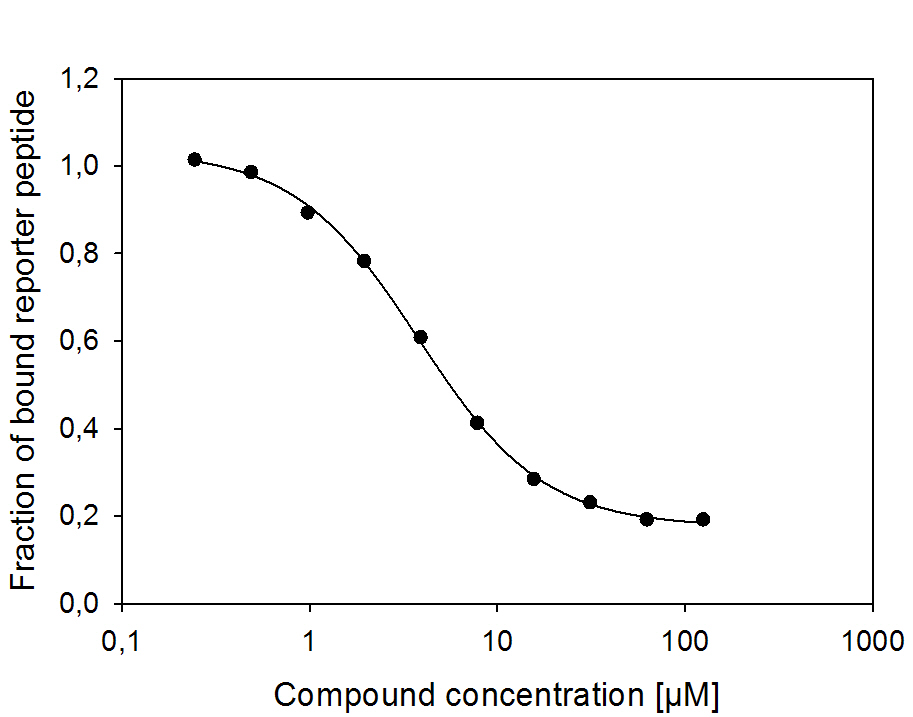  **18.** 1.5µM | 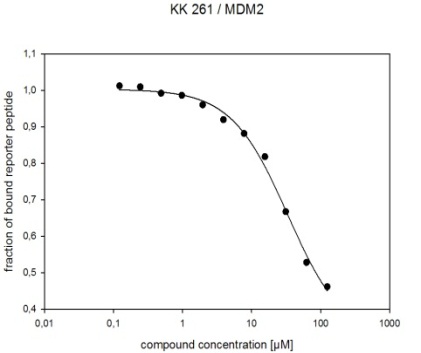  **21.** 11µM | **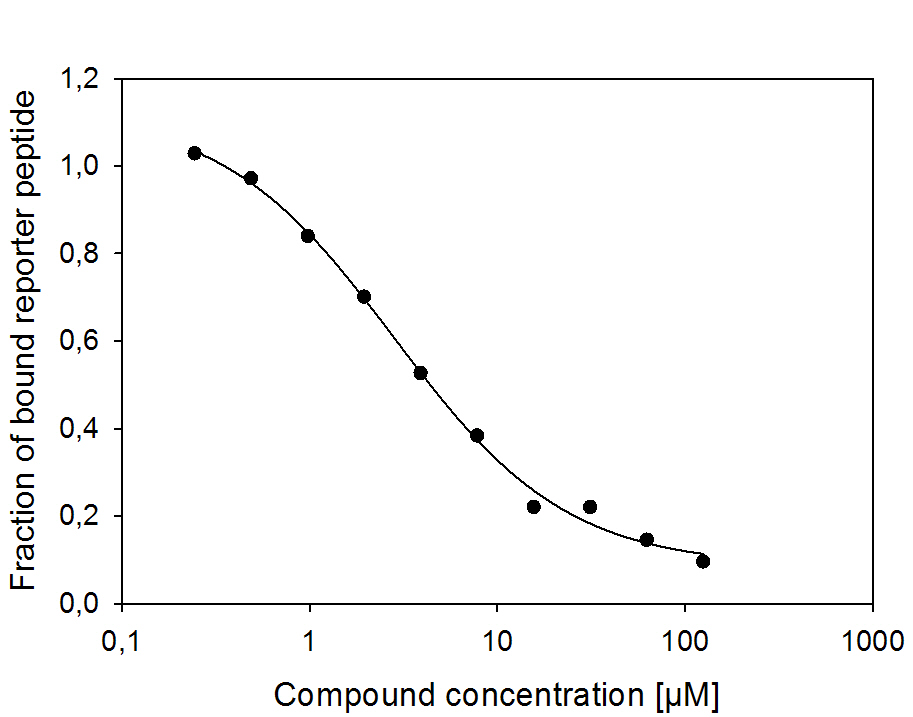**  **141.** 1.2µM |
|  | | |

Supplement: Table S2 — Inhibition curves of inhibitors from Figure 3(d) shown with rank and affinity. (DOC) [file pone.0032839.s004.doc]
