# Supplementary material for: Enabling Large-Scale Design, Synthesis and Validation of Small Molecule Protein-Protein Antagonists
Source: PLoS One. 2012 Mar 12;7(3):e32839. doi: 10.1371/journal.pone.0032839 (PMC3299697; doi:10.1371/journal.pone.0032839)
Supplement: Table S1 — Multicomponent reactions used in the generation of a tryptophan-biased library. These reactions, together with a set of roughly 1000 commercially available starting materials, define a theoretical chemical space of more than three trillion distinct chemical compounds. Requiring at least one indole starting material in each reaction yields as many as 190 billion compounds containing a tryptophan mimic. (DOC) [file pone.0032839.s003.doc]

| Reaction | Scheme | Reference |
| --- | --- | --- |
| 1 |  |  |
| 2 |  |  |
| 3 |  |  |
| 4 |  |  |
| 5 |  |  |
| 6 |  |  |
| 7 |  |  |
| 8 |  |  |
| 9 |  |  |
| 10 |  |  |
| 11 |  |  |
| 12 |  |  |
| 13 |  |  |
| 14 |  |  |
| 15 |  |  |
| 16 |  |  |
| 17 |  |  |
| 18 |  |  |
| 19 |  |  |
| 20 |  |  |
|  | | |
|  | | |
