# Supplementary material for: Enabling Large-Scale Design, Synthesis and Validation of Small Molecule Protein-Protein Antagonists
Source: PLoS One. 2012 Mar 12;7(3):e32839. doi: 10.1371/journal.pone.0032839 (PMC3299697; doi:10.1371/journal.pone.0032839)
Supplement: Methods S1 — Chemical Synthesis. (DOC) [file pone.0032839.s005.doc]

**Ethyl 3-(2-(benzylamino)-1-(((S)-1-methoxy-4-methyl-1-oxopentan-2-yl)amino)-2-oxoethyl)-6-chloro-1H-indole-2-carboxylate**

A mixture of ethyl 6-chloro-3-formyl-1*H*-indole-2-carboxylate (1mmol, 251mg), leucine (1mmol, 131mg), benzylisocyanide (1mmol, 117mg), in 10mL of methanol was stirred under RT overnight. The methanol was evaporated under reduced pressure. The residue was dissolved in ethyl acetate, and washed 2 times each with saturated sodium bicarbonate and saturated sodium chloride and dried over sodium sulfate. The ethyl acetate was evaporated under reduced pressure, and the residue was purified via chromatotron in 3:1 hexane/ethyl acetate to afford the final product as a yellow solid (102mg, yield 20%). HPLC-MS *t*_R_: 12.48, m/z [M+H]^+^: 513.9, [M-H]^+^:512.0. ^1^H NMR (600MHz, CDCl3) δ 0.480-0.496 (3H, d), 0.762-0.751 (3H, d), 1.352-1.382 (3H, t), 1.629-1.374 (3H, m), 3.220-3.191 (1H, m), 3.640 (1H, s), 4.361-4.254 (2H, m), 4.581-4.454 (2H, m), 5.281 (1H, s), 7.024-7.007 (1H, m), 7.130-7.128 (1H, d), 7.276-7.260 (2H, m), 7.376-7.305 (2H, m), 7.563 (1H, s), 7.637-7.622 (1H, d), 9.306 (1H, s). ^13^C NMR (600MHz, CDCl3) δ 14.2, 21.3, 22.9, 24.6, 42.2, 43.5, 51.8, 56.4, 57.7, 61.3, 111.9, 118.7, 120.4, 121.6, 122.5, 125.1, 127.4, 127.7, 127.9, 128.1, 128.6, 128.8, 131.7, 136.1, 138.4, 161.4, 171.7, 175.5

**(3-(2-(Benzylamino)-1-((1-(hydroxyamino)-4-methyl-1-oxopentan-2-yl)amino)-2-oxoethyl)-6-chloro-N-hydroxy-1H-indole-2-carboxamide (KK271)**

To 1 equivalent of Ugi product 10 equivalents of H_2_NOH HCl, 10 equivalents of NaOH, and 3 equivalents of Et_3_N, were added and let stir overnight at RT. Product precipitated out as a white solid (25mg, yield 95%). HPLC-MS *t*_R_: 17.29, m/z [M+H]^+^: 502.3, [M-H]^+^:500.34. ^1^H NMR (600MHz, MeOD) δ 0.54-0.53 (3H, d), 0.57-0.56 (1H, d), 0.67-0.66 (2H, d), 0.85-0.84 (3H, d), 0.93-0.90 (2H, m), 0.98-0.96 (4H, m), 2.02 (2H, s), 3.09-3.07 (1H, t), 3.69-3.68 (1H, d), 4.13-4.09 (2H, m), 4.33-4.28 (2H, m), 4.37-4.35 (3H, m), 4.46-4.43 (1H, d), 5.32 (1H, s), 7.01-6.98 (2H, m), 7.34-7.31 (2H, s), 7.46-7.45 (2H, s), 7.51 (1H, s), 7.81-7.73 (2H, m). ^13^C NMR (600MHz, MeOD) δ 6.5, 7.0, 7.8, 13.1, 19.5, 20.5, 20.6, 20.7, 20.9, 21.6, 21.8, 22.0, 22.1, 24.2, 24.3, 24.4, 41.1, 41.9,42.1, 42.5, 42.5, 42.6, 42.7, 42.9, 43.0, 51.0, 52.0, 54.5, 54.7, 55.7, 55.9, 56.2, 56.7, 57.6, 58.6, 60.1, 111.3, 111.6, 119.7, 119.8, 120.7, 121.0, 121.6, 122.0, 126.0, 126.5, 126.6, 126.6, 126.7, 126.8, 127.1, 127.4, 128.0, 128.0, 128.0, 128.2, 128.6, 128.7, 128.8, 129.0, 129.0, 129.9, 133.2, 133.3, 135.6, 136.1, 138.4, 138.5, 138.6, 140.2, 167.4, 167.5

**(6-Chloro-3-(4-(4-chlorobenzyl)-1-phenyl-1H-pyrazol-5-yl)-1H-indol-2-yl)((S)-3-((3-(dimethylamino)propyl) (methyl)amino)pyrrolidin-1-yl)methanone** **(1):**

HPLC/MS: *t*_R_ = 15.63 min, *m/z* = 629.3 [M+H]^+^ HRMS: C_35_H_39_Cl_2_N_6_O, [M+H]^+^; 629.2562 (calcd.), 629.2556 (found). ^1^H NMR (CD_3_OD, 600 MHz)**:** 0.80 (1H, m), 1.19 (3H, m), 1.45-1.73(1H, m), 2.05 (5H, m), 2.24-2.42(1H, m), 2.75-2.82 (10H, m), 3.09 (3H, w), 3.36-3.38 (1H, m), 3.40-3.63 (2H, m), 3.87-3.90 (2H, m), 5.24 (2H, m), 6.79 -6.99 (6H, m), 7.22-7.62 (7H, m), 9.07-9.08 (1H, m). ^13^C NMR (CD_3_OD, 150.92 MHz): 20.3, 24.2, 30.7, 39.0, 43.6, 43.6, 52.1, 52.8, 52.9, 55.3, 55.3, 101.2, 103.7, 113.5, 121.8, 123.4, 123.6, 123.7, 126.9, 128.0, 128.1, 129.8, 129.9, 130.4, 130.6,130.8, 131.0, 131.1, 131.9, 132.4, 133.6, 133.6, 133.9, 135.4, 135.6, 137.4, 137.8, 152.2, 153.5, 153.7, 153.8,153.9, 154.1, 162.4, 163.0, 169.3.

**(6-Chloro-3-(4-(4-chlorobenzyl)-1-(4-fluorophenyl)-1H-pyrazol-5-yl)-1H-indol-2-yl)((S)-3-((3-(dimethylamino) propyl)(methyl)amino)pyrrolidin-1-yl)methanone (2):**

HPLC/MS: *t*_R_ = 9.95 min, *m/z* = 647.4 [M+H]^+^

**Ethyl 3-(1-(N-benzylacetamido)-2-(benzylamino)-2-oxoethyl)-6-chloro-1H-indole-2-carboxylate (3):**

The mixture of ethyl 6-chloro-3-formyl-Boc-indole-2-carboxylate (0.2 mmol, 70.2 mg), benzylamine (0.2 mmol, 21.9 µL), benzyl isocyanide (0.2 mmol, 24.4 µL), acetic acid (0.2 mmol, 11.5 µL) in 0.5 mL of methanol was stirring under RT for 2 days. The Ugi product was purified by chromatography on silica gel (petroleum ether/ ethyl acetate, 3:1) as yellow solids (49 mg, yield: 40%). The Ugi product was treated with 0.5 mL of DCM, 100 µL of TFA stirring overnight under RT. After evaporation of the solvent, the product was purified by chromatography on silica gel (petroleum ether/ ethyl acetate, 1:2) as off-white solids (18 mg, yield: 44%). HPLC/MS: *t*_R_ = 10.97 min; *m/z* = 518.0 [M+H]^+^ HRMS: C_29_H_28_N_3_O_4_Cl, [M+Na]^+^; 540.1666 (calcd.), 540.1695 (found). ^1^H NMR (600 MHz, MeOD, major rotamer): 1.41 (t, 3H, J = 7.2 Hz), 2.23 (s, 3H), 4.33-4.45 (m, 6H), 4.85 (m, 1H), 6.28 (m, 1H), 6.60 (m, 1H), 6.81 (m, 1H), 6.95-6.99 (m, 4H), 7.19-7.25 (m, 5H), 7.31 (m, 1H), 7.57 (m, 1H). ^13^C NMR (150 MHz, MeOD, a mixture of rotamers): 13.3, 13.4, 20.8, 42.9, 43.2, 50.2, 54.4, 60.6, 60.8, 111.7, 111.8, 113.1, 113.5, 121.2, 121.3, 121.6, 124.5, 125.2, 125.5, 125.6, 126.2, 126.6, 126.8, 127.0, 127.3, 127.4, 127.8, 127.9, 128.0, 128.1, 130.38, 130.40, 136.4, 137.5, 138.1, 138.3, 160.5, 160.8.

**3-(1-(N-benzylacetamido)-2-(cyclohexylamino)-2-oxoethyl)-6-chloro-N-(2-methoxyethyl)-1H-indole-2-carboxamide (4):**

The mixture of ethyl 6-chloro-3-formyl-1H-indole-2-carboxylate (1 mmol, 251 mg), benzylamine (1 mmol, 110 µL), cyclohexyl isocyanide (1 mmol, 125 µL), acetic acid (1 mmol, 57.5 µL) in 0.5 mL of methanol was stirring under RT for 7 days. The Ugi product was purified by chromatography on silica gel (petroleum ether/ ethyl acetate, 3:1) as yellow solids (414 mg, yield: 81%). . The mixture of Ugi product (50.9 mg, 0.1 mmol), THF (1 mL), 4-(2-aminoethyl)pyridine (0.2 mmol, 23.7 uL), TBD (0.02 mmol, 3 mg) was stirring under 40 ^o^C overnight. The product was purified by chromatography on silica gel (methanol/ ethyl acetate, 1:5) as yellow solids (25 mg, yield: 43%). HRMS: C_33_H_36_ClN_5_O_3_Na, 608.2404 (calcd.), 608.2427 (found). ^1^H NMR (600 MHz, CDCl_3_): 0.85-1.34 (m, 6H), 1.57-1.92 (m, 7H), 2.03 (s, 3H), 3.04 (m, 2H), 3.74-3.90 (m, 3H), 4.75-4.90 (m, 2H), 5.80 (s, 1H), 6.52 (m, 2H), 6.86 (s, 1H), 6.95-7.02 (m, 4H), 7.20 (m, 2H), 7.32 (s, 1H), 7.49 (m, 1H), 8.42 (m, 2H), 8.57 (s, 1H), 10.11 (br.s, 1H). ^13^C NMR (150 MHz, CDCl_3_): 22.2, 24.7, 24.8, 24.9, 25.3, 29.7, 32.7, 33.0, 34.4, 40.4, 49.3, 51.5, 53.5, 108.3, 112.1, 121.5, 122.1, 124.2, 125.1, 125.2, 126.9, 128.2, 130.6, 131.3, 135.3, 136.9, 148.1, 149.7, 150.3, 160.8, 167.9, 174.3.

**6-Chloro-3-(4-(4-chlorobenzyl)-1-phenyl-1H-pyrazol-5-yl)-N-(7-(hydroxyamino)-7-oxoheptyl)-1H-indole-2-carboxamide (5):**

**6-Chloro-3-((4S,5R)-1-(4-chlorobenzyl)-4-phenyl-4-(2-(pyridin-4-yl)ethylcarbamoyl)-4,5-dihydro-1H-imidazol-5-yl)-N-(2-(pyridin-4-yl)ethyl)-1H-indole-2-carboxamide (6):**

HPLC/MS: *t*_R_ = 15.63 min, *m/z* = 629.3 [M+H]^+^

**Ethyl 6-chloro-3-(1-(N-(4-chlorobenzyl)formamido)-2-(cyclohexylamino)-2-oxoethyl)-1H-indole-2-carboxylate (7):**

The mixture of ethyl 6-chloro-3-formyl-1H-indole-2-carboxylate (0.2 mmol, 50.2 mg), 4-chlorobenzylamine (0.2 mmol, 24.4 µL), cyclohexyl isocyanide (0.2 mmol, 24.9 µL), formic acid (0.2 mmol, 7.9 µL) in 0.5 mL of methanol was stirring under RT for 7 days. The product was purified by chromatography on silica gel (petroleum ether/ ethyl acetate, 1:1) as yellow solids (55 mg, yield: 52%). HPLC/MS: *t*_R_ = 17.00 min; *m/z* = 530.1 [M+H]^+^ HRMS: C_27_H_29_N_3_O_4_Cl_2_Na, 552.1433 (calcd.), 552.1401 (found). ^1^H NMR (600 MHz, CDCl_3_, a mixture of rotamers): 0.86-1.07 (m, 6H), 1.31-1.39 (m, 9H), 1.58-1.87 (m, 10H), 3.65-3.84 (m, 3H), 4.25 (ABd, 1H, J = 15.0 Hz), 4.30-4.35 (m, 4H), 4.59 (ABd, 1H, J = 16.8 Hz), 4.85 (ABd, 1H, J = 15.6 Hz), 5.57 (m, 1H), 5.65 (m, 1H), 6.18 (s, 1H), 6.51 (m, 1H), 6.76 (s, 1H), 6.79-7.28 (m, 9H), 7.56 (m, 1H), 7.79 (m, 1H), 8.41 (s, 1H), 8.48 (s, 1H), 9.34 (s, 1H), 9.58 (s, 1H). ^13^C NMR (150 MHz, CDCl_3_, a mixture of rotamers): 14.3, 24.7, 24.8, 25.3, 25.4, 32.7, 32.8, 45.9, 49.0, 49.5, 52.1, 56.9, 61.7, 112.0, 112.4, 113.4, 114.9, 122.1, 122.5, 122.6, 124.7, 125.5, 126.4, 127.17, 127.23, 127.3, 127.8, 128.1, 128.2, 128.8, 129.1, 131.8, 131.9, 132.8, 135.86, 135.89, 136.0, 160.5, 160.6, 163.6, 164.6, 167.8, 168.0.

**7-(6-Chloro-3-(4-(4-chlorobenzyl)-1-phenyl-1H-pyrazol-5-yl)-1H-indole-2-carboxamido)heptanoic acid (8):**

HPLC/MS: *t*_R_ = 18.14 min, *m/z* = 589.4 [M+H]^+^

**Ethyl 6-chloro-3-(2-((4-chlorobenzyl)amino)-1-(((S)-1-methoxy-4-methyl-1-oxopentan-2-yl)amino)-2-oxoethyl)-1H-indole-2-carboxylate (10):**

A mixture of ethyl 6-chloro-3-formyl-1*H*-indole-2-carboxylate (1mmol, 251mg), leucine (1mmol, 131mg), *para*-chloro-benzylisocyanide (1mmol, 117mg), in 10mL of methanol was stirred under RT overnight. The methanol was evaporated under reduced pressure. The residue was dissolved in ethyl acetate, and washed 2 times each with saturated sodium bicarbonate and saturated sodium chloride and dried over sodium sulfate. The ethyl acetate was evaporated under reduced pressure, and the residue was purified via chromatotron in 3:1 hexane/ethyl acetate to afford the final product as a yellow solid (81mg, yield 15%). HPLC-MS *t*_R_:17.29, *m/z* [M+H]^+^: 549.8, [M-H]^+^:547.9 ^1^H NMR (600MHz, CDCl3) δ 0.51-0.49 (3H, d), 0.79-0.78 (3H, d), 1.40-1.38 (3H, t), 1.56-1.42 (3H, m), 3.22-3.19 (1H, m), 3.67 (1H, s), 4.38-4.35 (2H, m), 4.51-4.39 (2H, m), 5.27 (1H, s), 7.09-7.07 (1H, d), 7.18-7.17 (2H, d), 7.31-7.26 (2H, m), 7.41 (1H, s), 7.72-7.71 (1H, d), 8.98 (1H, s). ^13^C NMR (600MHz, CDCl3) δ 14.0, 21.4, 23.0, 24.5, 42.4, 42.9, 52.0, 56.2, 56.7, 62.3, 85.7, 115.2, 122.5, 123.3, 124.0, 125.1, 128.6, 128.8, 129.8, 133.1, 136.7, 148.6, 162.5, 170.0, 175.5.

**Ethyl 3-(2-(benzylamino)-1-(((S)-4-methyl-1-((2-morpholinoethyl)amino)-1-oxopentan-2-yl)amino)-2-oxoethyl)-6-chloro-1H-indole-2-carboxylate (18)**

A mixture of ethyl 6-chloro-3-formyl-1*H*-indole-2-carboxylate (1mmol, 251mg), leucine (1mmol, 131mg), benzylisocyanide (1mmol, 117mg), and 2-morpholinoethanamine (1mmol, 130mg) in 10mL of trifluoroethanol was stirred under RT overnight. The trifluoroethanol was evaporated under reduced pressure. The residue was dissolved in ethyl acetate, and washed 2 times each with saturated sodium bicarbonate and saturated sodium chloride and dried over sodium sulfate. The ethyl acetate was evaporated under reduced pressure, and the residue was purified via chromatotron in 1:1 hexane/ethyl acetate to afford the final product as a yellow solid (213mg, yield 35%). HPLC-MS *t*_R_:14.01, m/z [M+H]^+^: 612.0, [M-H]^+^:610.0. ^1^H NMR (600MHz, CDCl3) δ 0.82-0.81 (3H, d), 0.91-0.90 (3H, d), 1.40-1.37 (3H, t), 1.62-1.60 (1H, m), 1.76-1.74 (1H, m), 1.88-1.86 (1H, m), 2.86-2.85 (2H, m), 2.96-2.95 (1H, m), 3.08 (1H, m), 3.38-3.33 (2H, t), 3.63 (1H, s), 3.78-3.70 (3H, m), 3.94-3.85 (2H, m), 4.10-4.07 (1H, t), 4.29-4.25 (1H, m), 4.44-4.32 (3H, m), 6.14 (1H, s), 6.88 (1H, s), 7.03-7.01 (2H, m), 7.19-7.18 (1H, d), 7.21 (1H, s), 7.28 (1H, s), 7.52 (1H, s), 7.72-7.71 (1H, d), 8.94 (1H, s), 10.31 (1H, s). ^13^C NMR (600MHz, CDCl3) δ 13.8, 21.7, 21.9, 24.6, 33.9, 39.6, 44.2, 52.4, 53.1, 55.7, 57.4, 58.9, 62.9, 63.5, 69.5, 110.3, 113.0, 120.6, 123.5, 126.7, 127.1, 127.8, 128.7, 133.0, 136.2, 136.5, 161.7, 166.6.

**Ethyl 3-(2-(benzylamino)-1-(((S)-4-methyl-1-((3-morpholinopropyl)amino)-1-oxopentan-2-yl)amino)-2-oxoethyl)-6-chloro-1H-indole-2-carboxylate (21)**

A mixture of ethyl 6-chloro-3-formyl-1*H*-indole-2-carboxylate (1mmol, 251mg), leucine (1mmol, 131mg), benzylisocyanide (1mmol, 117mg), and 2-morpholinopropylamine (1mmol, 144mg) in 10mL of trifluoroethanol was stirred under RT overnight. The trifluoroethanol was evaporated under reduced pressure. The residue was dissolved in ethyl acetate, and washed 2 times each with saturated sodium bicarbonate and saturated sodium chloride and dried over sodium sulfate. The ethyl acetate was evaporated under reduced pressure, and the residue was purified via chromatotron in 1:1 hexane/ethyl acetate to afford the final product as a yellow solid (156mg, yield 25%)**.** HPLC-MS *t*_R_:14.99, *m/z* [M+H]^+^: 626.3, [M-H]^+^:624.7**.** ^1^H NMR (600MHz, CDCl3) δ 0.74-0.73 (3H, d), 0.89-0.88 (3H, d), 1.41-1.36 (3H, m), 1.58 (2H, s), 1.69-1.67 (1H, m), 1.87-1.86 (2H, m), 1.94-1.92 (2H, t), 3.15-3.13 (1H, m), 3.32-3.23 (4H, m), 3.46-3.42 (1H, d), 3.52-3.50 (1H, d), 3.59-3.56 (1H, m), 3.89-3.82 (4H, m), 4.03-4.00 (2H, m), 4.12-4.06 (3H, m), 4.27-4.24 (1H, m), 4.48-4.45 (1H, m), 5.87 (1H, s), 6.73-6.71 (1H, t), 7.05 (2H, m), 7.21 (1H, s), 7.22 (1H, s), 7.24 (1H, s), 7.28-7.27 (1H, m), 7.56-7.54 (3H, m), 8.13-8.12 (1H, m), 9.85 (1H, s).^13^C NMR (600MHz, CDCl3) δ 13.7, 21.0, 22.1, 22.8, 24.5, 27.6, 36.7, 39.9, 44.5, 52.2, 52.5, 55.0, 55.6, 58.5, 63.5, 63.7, 63.8, 63.8, 109.1, 112.1, 113.2, 114.0, 115.9, 117.8, 119.6, 124.1, 126.2, 127.4, 127.5, 128.0, 128.7, 128.8, 128.9, 133.7, 136.1, 136.2, 160.1, 160.3, 160.6, 160.9, 162.2, 166.1.
