# Supplementary material for: Enabling Large-Scale Design, Synthesis and Validation of Small Molecule Protein-Protein Antagonists
Source: PLoS One. 2012 Mar 12;7(3):e32839. doi: 10.1371/journal.pone.0032839 (PMC3299697; doi:10.1371/journal.pone.0032839)
Supplement: Methods S2 — Experimental Procedures and Supplementary References. (DOC) [file pone.0032839.s006.doc]

**Fluorescence polarization binding assays (FP).** All FP experiments were performed as described by Czarna et al. . Briefly, the fluorescence polarization experiments were read on an Ultra Evolution 384-well plate reader (Tecan) with the 485 nm excitation and 535 nm emission filters. The fluorescence intensities parallel (Int_parallel_) and perpendicular (Int_perpedicular_) to the plane of excitation were measured in parallel perpendicular black 384-well NBS assay plates (Corning) at room temperature (~20°C). The background fluorescence intensities of blank samples containing the references buffer were subtracted and steady-state fluorescence polarization was calculated using the equation: P = (Int_parallel_ - GInt _perpedicular_)/( Int_parallel_ + GInt _perpedicular_), and the correction factor G (G = 0.998 determined empirically) was introduced to eliminate differences in the transmission of vertically and horizontally polarized light. All fluorescence polarization values were expressed in millipolarization units (mP). The binding affinities of the fluorescent p53-derived peptide of Hu at al. (the P4 peptide in Czarna et al. ) towards MDM2 was determined in the buffer which contained 50 mM NaCl, 10 mM Tris pH 8.0, 1 mM EDTA, 10% DMSO. Competition binding assays were performed using the 10 nM fluorescent P4 peptide and 100 nM MDM2. Binding constant and inhibition curves were fitted using the SigmaPlot (SPSS Science Software).

**Protein expression, purification and crystallization.** Human MDM2 (residues 18-111) was cloned into the pET26 vector and expressed in the *Escherichia coli* BL21(DE3) Rosetta strain (Invitrogen). Cells were grown at 37°C and induced with 1 mM IPTG at an OD_600nm_ of 0.8 and grown for additional 4 h at 37°C. The recombinant protein expressed into inclusion bodies. The inclusion bodies were isolated by centrifugation of the bacterial lysate and washed with PBS containing 0.05% Triton-X100 and subsequently solubilized in 6 M GuHCl in 100 mM tris-HCl, pH 8.0, including 1 mM EDTA and 10 mM β-mercaptoethanol. The protein was then dialyzed against 4 M GuHCl, pH 3.5, 10 mM β-mercaptoethanol. For renaturation, the protein was diluted (1:100) into 10 mM Tris-HCl, pH 7.0, containing 1 mM EDTA and 10 mM β-mercaptoethanol, by adding the protein in several pulses into the refolding buffer. Refolding was performed overnight at 4°C. Following, ammonium sulphate was added to the final concentration of 1.5 M and after 3 h the sample was mixed with 10 ml of the butyl sepharose 4 Fast Flow (Pharmacia, FRG). The protein was eluted with 100 mM Tris-HCl, pH 7.2, containing 5 mM β-mercaptoethanol, and further purified on the HiLoad 16/60 Superdex200 gel filtration (Pharmacia) into buffer containing 5 mM Tris/HCl pH = 8.0, 50 mM NaCl, 10 mM β-mercaptoethanol. After addition of the molar excess of the small molecule inhibitor, KK271, the protein complex was concentrated to about 20 mg/ml and subjected to the crystallization screening with the sitting drop vapor diffusion method. The crystals of the MDM2-KK271 complex appeared after several days at 4^o^C in 0.2 M ammonium nitrate, 20% (w/v) PEG 3350 forming hexagonal bipyramides. All crystals were plunged frozen with 30% glycerol added to the mother liquor.

**Data collection and structure solution.** The dataset was collected on the SLS beamline PXII at Paul Scherrer Institut, Villingen, Switzerland. The collected data was indexed, integrated, scaled and merged with XDS and XSCALE .The MDM2-KK271 crystal belonged to P6_5_22 space group and diffracted to 2.15 Å. One complex containing one protein molecule and two inhibitor molecules was present in the asymmetric unit. Phase problem was solved by molecular replacement using the Molrep program from the CCP4 suite . MDM2 molecule from MDM2-nutlin cocrystal structure (1RV1; ) was used as a search model. The model was then subsequently improved by Arp/Warp and manually rebuild by iterative electron density fitting in MIFit program (http://code.google.com/p/mifit/) and refinement with Refmac5 . Ligand molecule and Refmac5 dictionary restraints were created using the dictionary module in MIFit. Water molecules were added by Arp/Warp solvent module. The electron densities for residues 18-24 and 109-111 were missing and thus, those residues were not included in the model. Additionally, several side-chains' atoms without clear electron density were omitted in the model. Data collection and refinement statistics are summarized below.

| Space group | P 6_5_ 2 2 |
| --- | --- |
| *Cell constant:* |  |
| a | 53.520 |
| b | 53.520 |
| c | 122.270 |
| Resolution range (Å) | 46.350 - 2.14 |
| Observed reflections | 63031 |
| Unique reflections | 11501 |
| *Whole resolution range:* |  |
| Completeness (%) | 97.6% |
| Rmerge | 2.6% |
| I/σ (I) | 32.03 |
| *Last resolution shell:* |  |
| Resolution range (Å) | 2.24-2.14 |
| Completeness (%) | 84.2% |
| Rmerge | 10.0% |
| I/σ (I) | 9.83 |
| *Refinement:* |  |
| No. of reflections | 10366 |
| Resolution (Å) | 18.467-2.140 |
| R factor (%) | 20.42 |
| R_free_ (%) | 22.79 |
| Average B (Å^2^) | 28.691 |
| rms bond length (Å) | 0.0098 |
| rms angles (°) | 1.317 |
| *Content of asymmetric unit:* |  |
| No. of complexes | 1 |
| No. of protein residues/atoms | 84/794 |
| No. of solvent atoms | 37 |
| PDB Data Bank ID code | XXXX |
| **Data collection and refinement statistics** | |

References

1. Czarna A*, et al.* (2009) High affinity interaction of the p53 peptide-analogue with human Mdm2 and Mdmx. (Translated from eng) *Cell Cycle* 8(8):1176-1184 (in eng).

2. Kabsch W (1993) Automatic processing of rotation diffraction data from crystals of initially unknown symmetry and cell constants. *Journal of Applied Crystallography* 26(6):795-800.

3. Vagin A & Teplyakov A (1997) MOLREP: an automated program for molecular replacement. *Journal of Applied Crystallography* 30(6):1022-1025.

4. Vassilev L*, et al.* (2004) In vivo activation of the p53 pathway by small-molecule antagonists of MDM2. *Science* 303(5659):844.

5. Lamzin VS & Wilson KS (1993) Automated refinement of protein models. *Acta Crystallographica Section D* 49(1):129-147.

6. Murshudov GN, Vagin AA, & Dodson EJ (1997) Refinement of Macromolecular Structures by the Maximum-Likelihood Method. *Acta Crystallographica Section D* 53(3):240-255.

7. Ugi I, Meyr R, Fetzer U, & Steinbrückner C (1959) Versammlungsberichte. *Angewandte Chemie* 71(11):373-388.

8. Dömling A & Ugi I (2000) Multicomponent Reactions with Isocyanides. *Angewandte Chemie International Edition* 39(18): 3168–3210.

9. Ugi I & Steinbrückner C (1960) Über ein neues Kondensations-Prinzip. *Angewandte Chemie* 72:267–268.

10. Subhas Chandra Pan & List B (2008) Catalytic Three-component Ugi Reaction. *Angewandte Chemie International Edition* 47(19):3622–3625.

11. Ugi I & Steinbrückner C (1961) Isonitrile, II. Reaktion von Isonitrilen mit Carbonylverbindungen, Aminen und Stickstoffwasserstoffsäure. *Chemische Berichte,* 94(3):734-742.

12. Dömling A, Beck B, & Magnin-Lachaux M (2006) 1-Isocyanomethylbenzotriazole and 2,2,4,4-tetramethylbutylisocyanide—cleavable isocyanides useful for the preparation of α-aminomethyl tetrazoles *Tetrahedron Lett.* 47(25):4289-4291.

13. Ugi I (1962) Neuere Methoden der präparativen organischen Chemie IV Mit Sekundär-Reaktionen gekoppelte α-Additionen von Immonium-Ionen und Anionen an Isonitrile. *Angewandte Chemie* 74:9-22.

14. Barrow JC*, et al.* (2008) Discovery and X-ray crystallographic analysis of a spiropiperidine iminohydantoin inhibitor of beta-secretase. (Translated from eng) *J Med Chem* 51(20):6259-6262 (in eng).

15. Ugi I, Demharter A, Hörl W, & Schmid T (1996) Ugi reactions with trifunctional α-amino acids, aldehydes, isocyanides and alcohols. *Tetrahedron* 52(26):11657-11664

16. Demharter A, Hör W, Herdtweck E, & Ugi I (1996) Synthesis of Chiral 1,1′-Iminodicarboxylic Acid Derivatives from α-Amino Acids, Aldehydes, Isocyanides, and Alcohols by the Diastereoselective Five-Center–Four-Component Reaction. *Angewandte Chemie International Edition* 35(2):173-175.

17. Ugi I (1962) The α-Addition of Immonium Ions and Anions to Isonitriles Accompanied by Secondary Reactions. *Angewandte Chemie International Edition* 1(1):8-21.

18. Dömling A, Kehagia K, & Ugi I (1995) Employment of a steroidal aldehyde in a new synthesis of β-lactam derivatives. *Tetrahedron* 51(35):9519-9522.

19. Harriman G (1997) Synthesi of small and medium sized 2,2-distributed lactams via the "intramolecular" three component Ugi reaction. *Tetrahedron Lett.* 38(32):5591-5594.

20. Short K, Ching B, & Mjalli A (1997) Exploitation of the Ugi 4CC reaction: Preparation of small molecule combinatorial libraries via soldi phase. *Tetrahedron* 53(19):6653-6697.

21. Nixey T, Kelly M, & Hulme C (2000) The one-pot solution phase preparation of fused tetrazole-ketopiperazines *tetrahedron Lett.* 41(45):8729-8733.

22. Chengzhi Z, Moran EJ, Woiwode TF, K.M. S, & A.M.M. M (1996) Synthesis of Tetrasubstituted Imidazoles via -(N-acyl-N-alkylamino)-β-ketoamides on Wang Resin. *Tetrahedron Lett.* 37:751-754.

23. Sungn K, Wu S-H, & Chen P-I (2002) Facile two-pot syntheses of novel alternating benzene/imidazole systems *Tetrahedron* 58(28):5599-5602.

24. Zhang C, Moran EJ, Woiwode TF, Short K, & Mjalli A (1996) Synthesis of Tetrasubstituted Imidazoles via α-(N-acyl-Nalkylamino)-β-ketoamides on Wang Resin. *Tetrahedron* 37(6):751-754.

25. Kazmaier U & Ackermann S (2005) A straightforward approach towards thiazoles and endothiopeptides via Ugi reaction. *Organic & Biomolecular Chemistry* 3:3184-3187.

26. Heck S & Dömling A (2000) A Versatile Multi-Component One-Pot Thiazole Synthesis. *Synlett* 2000(3):424-426.

27. Kolb J*, et al.* (2003) New MCRs: The first 4-component reaction leading to 2,4-disubstituted thiazoles. *Molecular diversity* 6(3-4):297-313.

28. Wang W, Joyner S, Khoury K, & Dömling A (2010) (−)-Bacillamide C: the convergent approach. *Organic & Biomolecular Chemistry* 8:529-532.

29. Illgen K*, et al.* (2004) A Versatile Synthesis of 6-Oxo-1,4,5,6-tetrahydro-pyrazine-2-carboxylic Acid Methyl Esters via MCR Chemistry. *Synlett* 2004(1):53-56.

30. Groebke K, Weber L, & Mehlin F (1998) Synthesis of Imidazo[1,2-a] annulated Pyridines, Pyrazines and Pyrimidines by a Novel Three-Component Condensation. *Synlett* 1998:661.

31. Bienayme H & Bouzid K (1998) A New Heterocyclic Multicomponent Reaction For the Combinatorial Synthesis of Fused 3-Aminoimidazoles. *Angewandte Chemie International Edition* 37:2234.

32. Hulme C & Lee Y-S (2008) Emerging approaches for the syntheses of bicyclic imidazo[1,2-x]-heterocycles. *Molecular diversity* 12(1):1-15.

33. Sun X, Janvier P, Zhao G, Bienaymé H, & Zhu J (2001) A Novel Multicomponent Synthesis of Polysubstituted 5-Aminooxazole and Its New Scaffold-Generating Reaction to Pyrrolo[3,4-b]pyridine. *Organic Lett.* 3(6):877–880.

34. Zhu J (2003) Recent Developments in the Isonitrile-Based Multicomponent Synthesis of Heterocycles. *European Journal of Organic Chemistry* 2003(7):1133-1144.

35. Tempest P*, et al.* (2001) Two-step solution-phase synthesis of novel benzimidazoles utilizing a UDC (Ugi/de-Boc/cyclize) strategystar, open. *Tetrahedron Lett.* 42(30):4959-4962

36. Richter HGF*, et al.* (2010) Discovery of novel and orally active FXR agonists for the potential treatment of dyslipidemia & diabetes *Bioorganic & Medicinal Chemistry Letters* In Press.

37. Hulme C, Ma L, Romano J, & Morrissette M (1999) Remarkable three-step-one-pot solution phase preparation of novel imidazolines utilizing a UDC (Ugi/de-Boc/cyclize) strategy *Tetrahedron Lett.* 40(45):7925-7928.

38. Hulme C*, et al.* (1998) Applications of N-BOC-diamines for the solution phase synthesis of ketopiperazine libraries utilizing a Ugi/De-BOC/Cyclization (UDC) strategy *Tetrahedron Lett.* 39(44):8047-8050.
